# Detection of Selection Signatures Underlying Production and Adaptive Traits Based on Whole-Genome Sequencing of Six Donkey Populations

**DOI:** 10.3390/ani10101823

**Published:** 2020-10-07

**Authors:** Zihui Zhou, Yingzhi Fan, Gang Wang, Zhenyu Lai, Yuan Gao, Fei Wu, Chuzhao Lei, Ruihua Dang

**Affiliations:** Key Laboratory of Animal Genetics, Breeding and Reproduction of Shaanxi Province, College of Animal Science and Technology, Northwest A&F University, Xianyang 712100, China; 15636110299@163.com (Z.Z.); w15029253912@126.com (Y.F.); wg18821714768@nwafu.edu.cn (G.W.); lzy18408210600@126.com (Z.L.); gaoyuan710@nwsuaf.edu.cn (Y.G.); 18392360732@163.com (F.W.); leichuzhao1118@126.com (C.L.)

**Keywords:** domesticated donkey, polymorphism, selection signatures, ZHp, di

## Abstract

**Simple Summary:**

After a long period of artificial selection, the donkey now presents a variety of body types and production performance values. In this experiment, we performed selective signal scanning on the second-generation resequencing data of six different varieties. The regions and candidate genes related to artificial selection were identified to provide reference for future breeding.

**Abstract:**

Donkeys (*Equus asinus*) are an important farm animal. After long-term natural and artificial selection, donkeys now exhibit a variety of body sizes and production performance values. In this study, six donkey breeds, representing different regions and phenotypes, were used for second-generation resequencing. The sequencing results revealed more than seven million single nucleotide variants (SNVs), with an average of more than four million SNVs per species. We combined two methods, Z-transformed heterozygosity (ZHp) and unbiased estimates of pairwise fixation index (di) values, to analyze the signatures of selection. We mapped 11 selected regions and identified genes associated with coat color, body size, motion capacity, and high-altitude adaptation. These candidate genes included staining (*ASIP* and *KITLG*), body type (*ACSL4*, *BCOR*, *CDKL5*, *LCOR*, *NCAPG*, and *TBX3*), exercise (*GABPA*), and adaptation to low-oxygen environments (*GLDC* and *HBB*). We also analyzed the SNVs of the breed-specific genes for their potential functions and found that there are three varieties in the conserved regions with breed-specific mutation sites. Our results provide data to support the establishment of the donkey SNV chip and reference information for the utilization of the genetic resources of Chinese domestic donkeys.

## 1. Introduction

The donkey (*Equus asinus*) is an important livestock species used for farming that originates from about 5000 years ago. Therefore, the donkey has been a witness to the progress of human history and civilization. However, with the increasing use of modern agricultural machinery and efficient transportation, the value of the draft donkey has gradually declined, but the value of donkey meat and milk has gradually increased. Factors such as diverse ecological changes, human migration, and socio-economic impacts have influenced the phenotypic characteristics of donkeys; there are now 157 registered donkey varieties. The research of selection signature plays an important role in animal breeding and population evolution. As the cost of single nucleotide polymorphism arrays (e.g., SNV (single nucleotide variant) chips) and high-throughput sequencing continues to decrease, research on the selection signals of domesticated animals is becoming more common. For example, selection signal screens have been conducted in domesticated pigs [1,2,3], chickens [4], cattle [5,6,7], sheep [8,9], goats [10,11,12], and dogs [13]. Several statistical methods have also been developed to detect the genomic regions related to selection in domesticated animals, such as overall low heterozygosity [3,4], genetic diversity patterns [14], haplotype homozygosity [15], and integrated haplotype score (|iHS|) [16]. However, there have been few studies on the selection signals of Chinese domestic donkeys.

**Figure 1:**
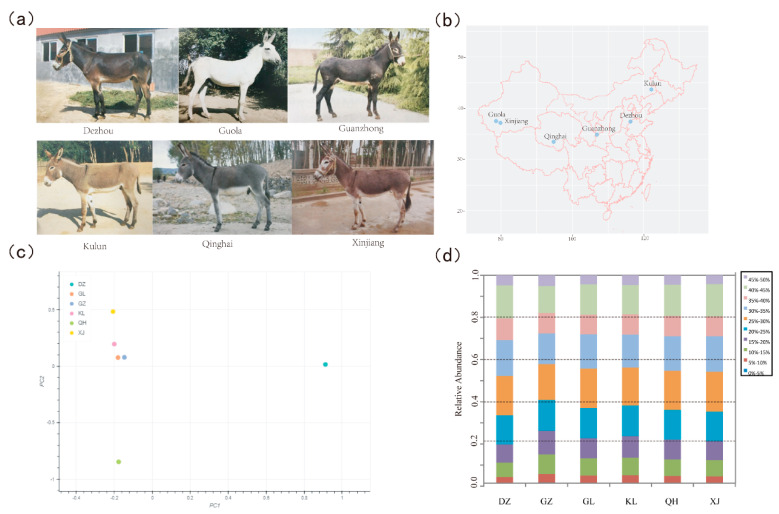
Summary of six donkey breeds. (**a**) The six donkey breeds included in this study. (**b**) Geographic map indicating the distribution of the donkeys sampled in this study. Each point represents the location of sampling. The map was generated using the ‘ggmap’ package in R (version 3.5.2) (R Core Team 2018). (**c**) Principal components analysis (PCA) of six donkey breeds using the PC1 and PC2 components. DZ: Dezhou; GL: Guola; GZ: Guanzhong; KL: Kulun; QH: Qinghai; and XJ: Xinjiang. (**d**) A schematic representation of minor allelic frequency (MAF) plotted as a function of distance for each donkey population.

The purpose of this study was to combine whole-genome resequencing technology with selective scavenging analysis to study the selected regions and genes unique to six Chinese donkey breeds, as well as to reveal the molecular mechanisms of their germplasm formation. The selection signatures were investigated by the pool sequencing of six distinct donkey populations (Table S1), including a black coated breed (Dezhou donkey), a highland breed (Qinghai donkey), two large donkey breeds (Hetian Qing donkey (Guola donkey) and Guanzhong donkey), and two donkey breeds with a small body size (Kulun donkey and Xinjiang donkey) (Figure 1a,b). The obtained SNVs were analyzed by calculating the Z-transformed heterozygosity (ZHp) and di (unbiased estimates of pairwise fixation index) values. Our results contribute to a better understanding of the genomic features donkey selection and reveal the genomic regions and genes controlling the production and adaptation of traits.

## 2. Materials and Methods

### 2.1. Ethics Approval

We performed the experiment according to the Regulations of the Administration of Affairs Concerning Experimental Animals (Ministry of Science and Technology, Beijing, China, 2004), and our research was approved by the Institutional Animal Care and Use Committee of Northwest A&F University.

### 2.2. Animals and Whole Genome Sequencing

Approximately 10 animals from each breed were combined into a pool for high-throughput resequencing (Table S1). According to the improved phenol–chloroform method [17], the genomic DNA of 57 individuals, representing 6 breeds of donkeys (Dezhou donkey = 9; Qinghai donkey = 9; Guola donkey = 10; Guanzhong donkey = 10; Kulun donkey = 10; and Xinjiang donkey = 9) were extracted from donkeys’ blood. These donkeys are not related to each other up to three generations. Six paired-end libraries (insert sizes: 180, 300, and 500 bp) were constructed and sequenced on the Illumina HiSeq Xten platform (2×150 bp) at Novogene (https://www.ncbi.nlm.nih.gov/biosample/13284254, https://www.ncbi.nlm.nih.gov/biosample/13284255, https://www.ncbi.nlm.nih.gov/biosample/13284256, https://www.ncbi.nlm.nih.gov/biosample/13284257, https://www.ncbi.nlm.nih.gov/biosample/13284258, and https://www.ncbi.nlm.nih.gov/biosample/13284259). Library preparation and sequencing followed the manufacturer’s instructions. Variant detection was conducted with the Genome Analysis Toolkit (GATK-3.8, https://github.com/broadinstitute/gatk/). To filter the SNVs for flowing analysis, we required at least three reads with different start sites supporting the non-reference allele.

### 2.3. Reads Alignment and Variations Calling

Reads were aligned to the donkey reference genome ASM130575v1 (https://www.ncbi.nlm.nih.gov/assembly/GCF_001305755.1/) [18] using BWA (0.7.13-r1126 version), then SAMtools (https://github.com/samtools/samtools/releases/) was used to sort the generated BAM files, and these were finally followed by duplicate removal using Picard-Tools-2.16.0 (http://broadinstitute.github.io/picard/). The Genome Analysis Toolkit (GATK-3.8) [19] was utilized to perform local realignment around existing indels and basic quality score recalibration. To ensure the accuracy of the SNV information, strict detection conditions were set: (1) there was only one mutation type, (2) the minimum quality value was ≥ 30, and (3) the minimum coverage was ≥ 68. The SNVs were formatted and functionally annotated using the SnpEff software and the gff (general feature format) annotation file of the donkey reference genome. Through the vcf (variant call format) file obtained in the previous step, principal component analysis (PCA) was performed with the PLINK and smartPCA software to evaluate the genetic relationships between breeds. The minor allelic frequency (MAF) was calculated using the VCFTOOLS software, and the distribution of the MAF was viewed to determine the quality of the SNP.

### 2.4. Detection of Selected Loci

To identify the regions under selection, the Z-transformed heterozygosity (ZHp) approach was utilized, as previously described [1,2,3,4]. To calculate the results, the PLINK software was used. Briefly, in an overlapping sliding window, Hp was calculated as:

(1) $$Fst=\frac{MSP-MSG}{MSP+\left(n-1\right)MSG}$$

where Σnmaj is the sum of major allele frequencies and ΣnMin is the sum of the MAF within each window. Individual Hp values were Z-transformed as:

(2) $$ZHp=\frac{\left(Hp-\mu Hp\right)}{\sigma Hp}$$

where *μHp* is the overall average heterozygosity and *σHp* is the standard deviation for all windows in each subpopulation. To detect putative selected loci in this study, we first calculated the pooled heterozygosity (Hp) and its Z-transformations, *ZHp*, in sliding 50 KB windows along the scaffolds [20,21]. Putatively selected loci were defined as genetic regions in overlapping windows with extremely low ZHp values (<−7) and extremely high di values (top 1%).

### 2.5. Bioinformatics Analysis of Breed Specific SNVs

After obtaining the selected genes, we identified breed-specific genes and analyzed the genes and their SNVs within 1000 bp upstream and downstream. We selected and downloaded the gene sequences of 11 mammals (*Bos taurus*, *Ovis aries*, *Capra hircus*, camels, *Felis catus*, *Canis lupus familiaris*, *Equus caballus*, *Homo sapiens*, *Sus scrofa*, whales, and dolphins) and the donkey. We determined the conserved regions of these genes by sequence alignment, and we analyzed the SNVs in these regions to predict breed-specific SNVs that may have certain functions.

## 3. Results

### 3.1. Genome Resequencing of Six Donkey Breeds

We utilized six genetically and phenotypically diverse domestic donkey breeds to systematically analyze the signatures of selection (Figure 1b; Table S1). Whole-genome sequencing (WGS) [18] was performed via Chicago HiRise [22] on an Illumina HiSeq Xten platform using pooled DNA from each breed. Genome sequencing yielded a total of 202 GB of raw data and produced 205–246 million sequence reads per breed (Table 1).

Over 99.8% of the generated sequence reads mapped to approximately 98.22% (98.18–98.38%) of the newly annotated donkey reference genome (ASM130575v1), thus indicating high-quality sequencing results. The sequence coverage averaged 11.5× per breed within a range of 10- to 12-fold. single nucleotide variants (SNVs) varied from 4.3 to 4.6 million in each population (Table 2).

PLINK and smartPCA were used to perform the principal component analysis (PCA) on the SNVs. The PCA results (shown in Figure 1c) classified the six donkey breeds according to their genetic differentiation. The Guanzhong donkey and Guola donkey were clustered together and close to the Kulun donkey, while the other three breeds were separate. This clustering pattern was consistent with the findings of a prior study on the genetic diversity of donkey breeds in China [23].

### 3.2. Identification of Coding SNVs and Short Insertions/Deletions

After the data were filtered, there were more than four million confirmed SNVs in each breed. Around 88–93% of the SNVs were heterozygous, and the Guanzhong, Guola, and Kulun breeds had the largest frequency of heterozygous SNVs. This suggests that these three breeds have not undergone recent intensive selection. The SNVs were rarely located in coding regions (~1.4%). Furthermore, non-synonymous and synonymous variants were identified in the donkey genome (Table 2); there were more synonymous variants than non-synonymous substitutions. The frequency of non-synonymous SNVs in each breed was stable (~47.1%; Figure S1) and is close to the frequency of non-synonymous SNVs in the horse [24]. SNVs of large impact were also identified in the donkey genome (premature stop codons, start codon to non-start codon, stop codon to non-stop, and splice site; Figure S2).

We observed the distribution of minor allelic frequency (MAF) with 10 continued classes from 0–0.05 to 0.45–0.50 for each breed (Figure 1d). The largest group of SNVs had an MAF in the range of 0.25–0.30 (16–18%), whereas the proportion of rare alleles (MAF < 0.05) accounted for <0.5% of the total SNVs (most rare alleles were removed during the SNV calling process).

### 3.3. Identification of Selected Loci and Candidate Genes

**Figure 2:**
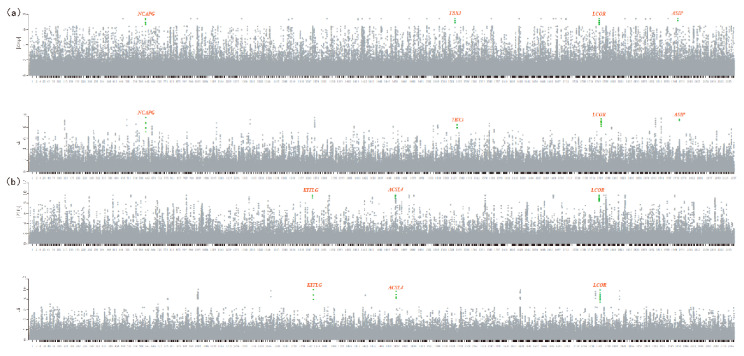
Overview of selective sweeps in the Dezhou and Xinjiang breeds plotted by Z-transformed heterozygosity (ZHp) and unbiased estimates of pairwise fixation index (di) values. (**a**) Dezhou donkey breed. (**b**) Xinjiang donkey. Functional genes are highlighted, and red and bold characters represent overlapping genes that were generated using the two methods. Absolute values of ZHp were used for plotting.

**Figure 3:**
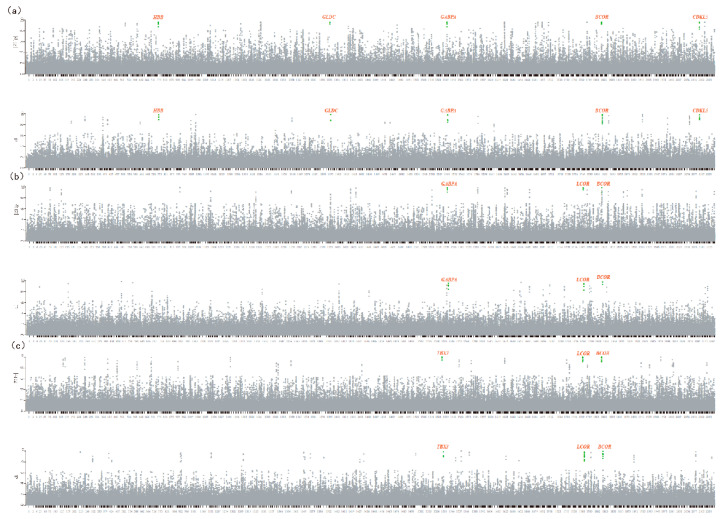
Overview of selective sweeps in the Qinghai, Guola, and Kulun donkeys plotted by ZHp and di values. (**a**) Qinghai breed. (**b**) Guola breed. (**c**) Kulun breed.

The ZHp value was calculated in sliding 50 KB windows along the scaffolds of sequence reads corresponding to the most and least frequently observed alleles at all SNV positions [3,4]. A total of 234,063 windows was obtained, and the ZHp and di values of each group’s sliding window were calculated (Figure 2 and Figure 3). Only the windows with more than 10 SNVs were used in the analyses. The selected regions and genes are marked in the figure. To evaluate population differentiation among specific breeds, the genetic differentiation between every pairwise comparison was measured using the fixation index (Fst), and di values were calculated [25]. Putatively selected loci were defined as genetic regions in overlapping windows with extremely low ZHp values (<−7) and extremely high di values (top 1%). Two selection signal analysis methods were used to determine the overlap region and find the candidate genes in the ZHp value upstream and di value downstream regions. The number of candidate genes identified in Dezhou, Quola, Qinghai, and Xinjiang donkeys was 4, 3, 5, and 3, respectively (Table 3).

## 4. Discussion

Pool sequencing is an efficient, low-time, and low-cost method for quickly and efficiently finding candidate gene regions associated with target traits using bulk segregant analysis (BSA). At present, many articles similar to our research have also chosen this method [22,26,27].

### 4.1. Coat Color

A total of 18 genomic regions were found within the top 1% (di > 7), and a list of candidate genes was generated (*KITLG* is also included in the list). By analyzing the heterozygosity of the Xinjiang breed, 26 distinct loci were identified (ZHp < −7), including the well-known coat color gene *KITLG* (Figure 2a; Tables S1 and S2). *KITLG*, which encodes for the ligand of c-Kit, plays a role in the melanocyte production pathway. *KITLG* variants cause different coat color phenotypes such as roan. The migration and survival of melanocytes derived from neural crest cells depend on the normal expression of the *KIT* gene and the presence of the mast cell growth factor/stem cell growth factor (MGF/SGF). If *KIT* is mutated or the *SGF* is absent, the melanocytes will not survive, and the resulting lack of melanocytes and melanin particles will produce a white coat color [28,29]. Variants at *KIT* are known to be associated with depigmentation or leucism.

Four loci overlapped between the genetic regions with the lowest ZHp and highest di values. One of the overlapping regions contained the strongest candidate gene for coat color (*ASIP*) in the Dezhou breed (Table 3). The *ASIP* gene can affect the production of brown melanin in several ways, and it has been implicated as a strong candidate controlling the coat color patterns of horses and donkeys [26,30,31]. Related studies have also shown that the *ASIP* gene is associated with the coat color phenotype of Dezhou donkeys [32,33].

### 4.2. Body Size

Of the six donkey breeds, the body sizes of the Dezhou and Guola donkeys were the largest; the average weight of the Dezhou and Goula donkeys was more than 200 kg, but the bodyweight of the smallest variety, the Qinghai donkey, was found to be only 110 kg for males and 120 kg for females. Advances in agriculture have gradually decreased the value of donkeys as servants, but the consumption of meat has gradually increased. Thus, the development of genetic breeding programs for donkey meat is particularly important.

Six of the 11 selected genes identified in this study were related to body size, namely *TBX3*, *NCAPG*, *LOCR*, *BCOR*, *CDKL5*, and *ACSL4*. These selected genes have also been reported in other species. SNV comparisons of the Debao pony, the Yili horse, and the Mongolian horse revealed that *TBX3* was significantly associated with body size, and *ACSL4* and *CDKL5* were associated with growth traits.

### 4.3. Motion Capacity and High-Altitude Adaptation

Of the five breeds, the Guola and Qinghai breeds are the major livestock species that serve as sources of transportation for Xinjiang and Qinghai inhabitants. Both breeds have a strong motion capacity even though the Qinghai breed lives in a mountainous environment. An analysis of the sequence heterozygosity and divergence of the breeds revealed overlapping regions with the lowest ZHp and highest di values (ZHp < −9 and di > 9). These regions contained the strongest candidate gene, *GABPA*, which has also been identified in horses. Schroder et al. performed a genome-wide association study (GWAS) with the jumping ability of Hanover’s warm-blooded horse and found an association with the sport-related gene *GABPA* [32].

The Qinghai donkey is well-adapted to high altitudes between 3000 and 5000 m. In the genome of the Qinghai donkey, 31 loci were identified as selected regions based on the calculated ZHp values (Figure 3b; Tables S1 and S2), and a total of 24 regions were within the top 1% of the distribution (di > 7). We identified two regions that exhibited the strongest selection signature by an overlap of the genomic regions with high di and low ZHp values. Two genes within these regions were previously implicated in the adaptation of Tibetan dwellers. The *HBB* gene is associated with the high-altitude adaptation of Tibetan people [33], and the *GLDC* gene in Tibetan pigs represents a genetic adaptation for the low oxygen concentrations of high-elevation environments [34].

**Figure 4:**
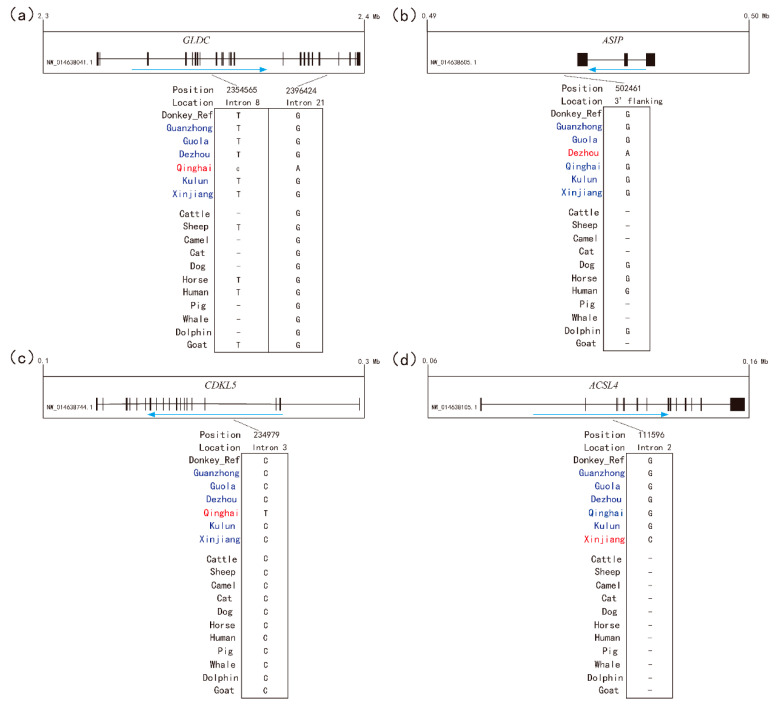
The conservation analyses identified candidate functional mutations that are specific to donkey breeds. Breed-specific SNVs within or close to the key selected genes. (**a**) *GLDC*, (**b**) *ASIP*, (**c**) *CDKL5*, and (**d**) *ACSL4* were screened, and the SNVs localized to evolutionary conserved sites were retained. The locations and positions of the candidate SNVs are indicated.

We further analyzed the functional importance of the SNVs in the seven breed-specific selected genes (*ASIP*, *CDKL5*, *GLDC*, *HBB*, *KITLG*, *ACSL4*, and *NCAPG*). The breed-specific SNVs in four of these genes (*GLDC*, *ASIP*, *CDKL5*, and *ACSL4*) are in evolutionarily conserved regions (Figure 4a–d), indicating that these SNVs may have certain functions. Consistent with a previous finding in goat domestication [27], none of these conserved SNVs were located in coding regions that lead to amino acid exchanges. This indicates that the genetic basis of donkey production and adaptive traits is complex and likely involves regulatory variants.

## 5. Conclusions

In this experiment, ZHp and di values were used to determine the genetic regions under selection in five domestic donkey breeds. Overlapping areas were identified and annotated, revealing a total of 11 selected genes. These genes are involved in hair color, body size, motion capacity, and adaptations for low-oxygen environments. Through the analysis of the mutation sites of each selected gene, possible functional breed-specific SNVs were also identified and revealed potential mechanisms of differentiation among several cultivars of Chinese domestic donkeys. In the age of large volume data from genomics and bioinformatics, the technology of whole-genome sequencing provides a new method to characterize the domestic donkey and provides a reference for future breeding practices.

## Figures and Tables

**Table 1:** Number of reads.

Breed	Raw Data (G)	Clean Data (G)	Reads Number (M)	Reads for Alignment (%)	Sequence Coverage
DZD	33.30	33.19	214.63	214.38(99.88)	11.23×
GLD	31.70	31.60	210.92	210.71(99.90)	11.15×
GZD	37.12	36.99	246.83	246.57(99.89)	12.88×
KLD	33.86	33.76	225.34	225.11(99.90)	11.81×
QHD	32.09	31.95	213.04	212.82(99.90)	11.18×
XJD	34.07	33.93	205.54	205.30(99.88)	10.74×

Note: DZD, Dezhou donkey; GLD, Guola donkey; GZD, Guanzhong donkey; KLD, Kulun donkey; QHD Qinghai donkey; and XJD, Xinjiang donkey.

**Table 2:** Summary and annotation of single nucleotide variants (SNVs) and indels in the donkey genome.

Breed	Number of Animals	SNVs Number	SNVs		Coding SNVs	
			Homozygote	Heterozygote (%)	Nonsynonymous Mutation (%)	Synonymous Mutation
DZD	9	4,372,377	490,631	3,881,746(88.78)	21,996(47.76)	24,057
GLD	10	4,548,452	336,032	4,212,420(92.61)	23,036(46.55)	26,449
GZD	10	4,658,120	320,497	4,337,623(93.12)	23,446(47.91)	25,489
KLD	10	4,607,547	339,569	4,267,978(92.63)	22,821(47.51)	25,213
QHD	9	4,480,050	390,000	4,090,050(91.29)	23,589(46.0)	27,688
XJD	9	4,455,917	397,306	4,058,611(91.08)	22,963(46.96)	25,937

Note: DZD, Dezhou donkey; GLD, Guola donkey; GZD, Guanzhong donkey; KLD, Kulun donkey; QHD Qinghai donkey; and XJD, Xinjiang donkey.

**Table 3:** Overlapping genes identified by both ZHp and di for different donkey breeds.

Candidate Gene	Scaffold	Annotation	ZHp	di
Dezhou donkey				
*TBX3*	NW_014638181.1	T-box 3	−7.34	8.55
*NCAPG*	NW_014637278.1	non-SMC (spondylo-metaphyseal chondrodysplasia) condensin I complex subunit G	−7.34	9.77
*LCOR*	NW_014638419.1	ligand dependent nuclear receptor corepressor	−7.34	9.60
*ASIP*	NW_014638605.1	agouti signaling protein	−7.20	9.50
Guola donkey				
*BCOR*	NW_014638463.1	B-cell CLL/lymphoma 6 corepressor	−9.90	9.89
*GABPA*	NW_014638188.1	GA binding protein transcription factor subunit alpha	−9.90	9.66
*LCOR*	NW_014638419.1	ligand dependent nuclear receptor corepressor	−9.90	9.54
Kulun donkey				
*BCOR*	NW_014638463.1	B-cell CLL/lymphoma 6 corepressor	−9.98	9.95
*TBX3*	NW_014638181.1	T-box 3	−9.98	9.89
*LCOR*	NW_014638419.1	ligand dependent nuclear receptor corepressor	−9.98	9.88
Qinghai donkey				
*GABPA*	NW_014638188.1	GA binding protein transcription factor subunit alpha	−9.60	9.86
*CDKL5*	NW_014638744.1	cyclin dependent kinase like 5	−9.60	9.85
LOC106830211(*HBB*)	NW_014637421.1	hemoglobin subunit beta	−9.60	9.88
*BCOR*	NW_014638463.1	B-cell CLL/lymphoma 6 corepressor	−9.60	9.98
*GLDC*	NW_014638041.1	glycine decarboxylase	−9.60	9.98
Xinjiang donkey				
*KITLG*	NW_014638016.1	Receptor tyrosine kinase(KIT) ligand	−9.60	9.95
*LCOR*	NW_014638419.1	ligand dependent nuclear receptor corepressor	−9.60	9.88
*ACSL4*	NW_014638105.1	acyl-CoA synthetase long chain family member 4	−9.60	9.60

## Supplementary Materials

The following are available online at https://www.mdpi.com/2076-2615/10/10/1823/s1, Table S1: Pooled sample information for resequencing data from donkeys. Donkey DNA pools represent six separate breeds. The number of individuals from each breed (n) and the average sequence and assembly coverage per pool are indicated. Table S2: The number of candidate regions and genes identified by ZHp and di in different breeds. Figure S1: The number of synonymous and non-synonymous mutations in each donkey breed. DZ: Dezhou; GL: Guola; GZ: Guanzhong; KL: Kulun; QH: Qinghai; and XJ: Xinjiang. Figure S2: The number of SNVs with large effects (premature stop, stop codon to nonstop codon, start codon to non-start codon, and splicing sites) in each donkey breed. DZ: Dezhou; GL: Guola; GZ: Guanzhong; KL: Kulun; QH: Qinghai; and XJ: Xinjiang.

## Abbreviations

SNVs	single nucleotide variants
di	unbiased estimates of pairwise fixation index
ZHp	Z-transformed heterozygosity
WGS	Whole-genome sequencing
PCA	principal component analysis
MAF	minor allelic frequency
Fst	fixation index
