# Supplementary material for: Detection of Selection Signatures Underlying Production and Adaptive Traits Based on Whole-Genome Sequencing of Six Donkey Populations
Source: Animals (Basel). 2020 Oct 7;10(10):1823. doi: 10.3390/ani10101823 (PMC7600737; doi:10.3390/ani10101823)
Supplement: Supplementary file 1 [file animals-10-01823-s001.zip › animals-929465-suppl/SupMaterial/Table.DOCX]

**Supplementary Table S1** Pooled samples information for resequencing data from donkeys. Donkey DNA pools represent six separate breeds respectively. The number of individuals from each breed (n) and the average sequence and assembly coverage per pool are indicated.

| **Breed** | **No. of samples** | **Sequence coverage(x)** | **Phenotype/Feature** |
| --- | --- | --- | --- |
| Dezhou | (F=9) | 11.23 | Black coated, higher body weight |
| Hetian Gray (Guola) | (F=10) | 11.15 | Grey coated, higher body weight |
| Guanzhong | (F=10) | 12.88 | Higher body weight |
| Kulun | (F=10) | 11.81 | Lower body weight |
| Qinghai | (F=9) | 11.18 | Highland adaption, lower body weight |
| Xingjiang | (F=9) | 10.74 | Lower body weight |

**Supplementary Table S2** The number of candidate regions and genes identified by ZHp and di in different breeds.

| **Breed** | **ZHp** | **di** | **Overlapped regions** | **Overlapped genes** |
| --- | --- | --- | --- | --- |
| Dezhou | 37 | 26 | 4 | 4 |
| Guola | 30 | 26 | 3 | 3 |
| Qinghai | 31 | 24 | 5 | 5 |
| Xinjiang | 26 | 18 | 3 | 3 |
| Kulun | 33 | 29 | 3 | 3 |
| Total | 98 | 94 | 11 | 11 |
