# Supplementary figures and images for: Detection of Selection Signatures Underlying Production and Adaptive Traits Based on Whole-Genome Sequencing of Six Donkey Populations
Source: Animals (Basel). 2020 Oct 7;10(10):1823. doi: 10.3390/ani10101823 (PMC7600737; doi:10.3390/ani10101823)

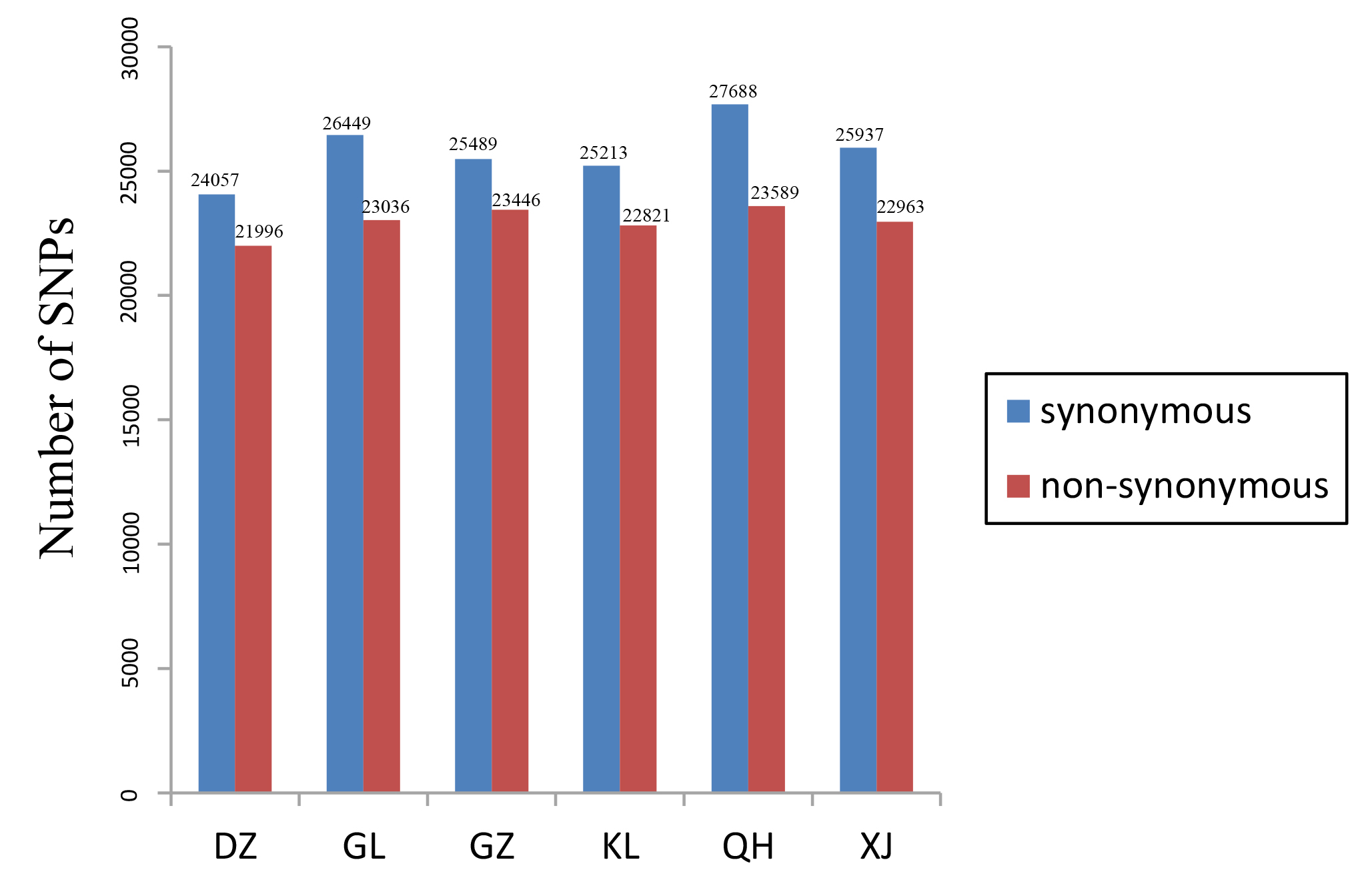

Supplement: Supplementary file 1 [file animals-10-01823-s001.zip › animals-929465-suppl/SupMaterial/S1.JPEG]

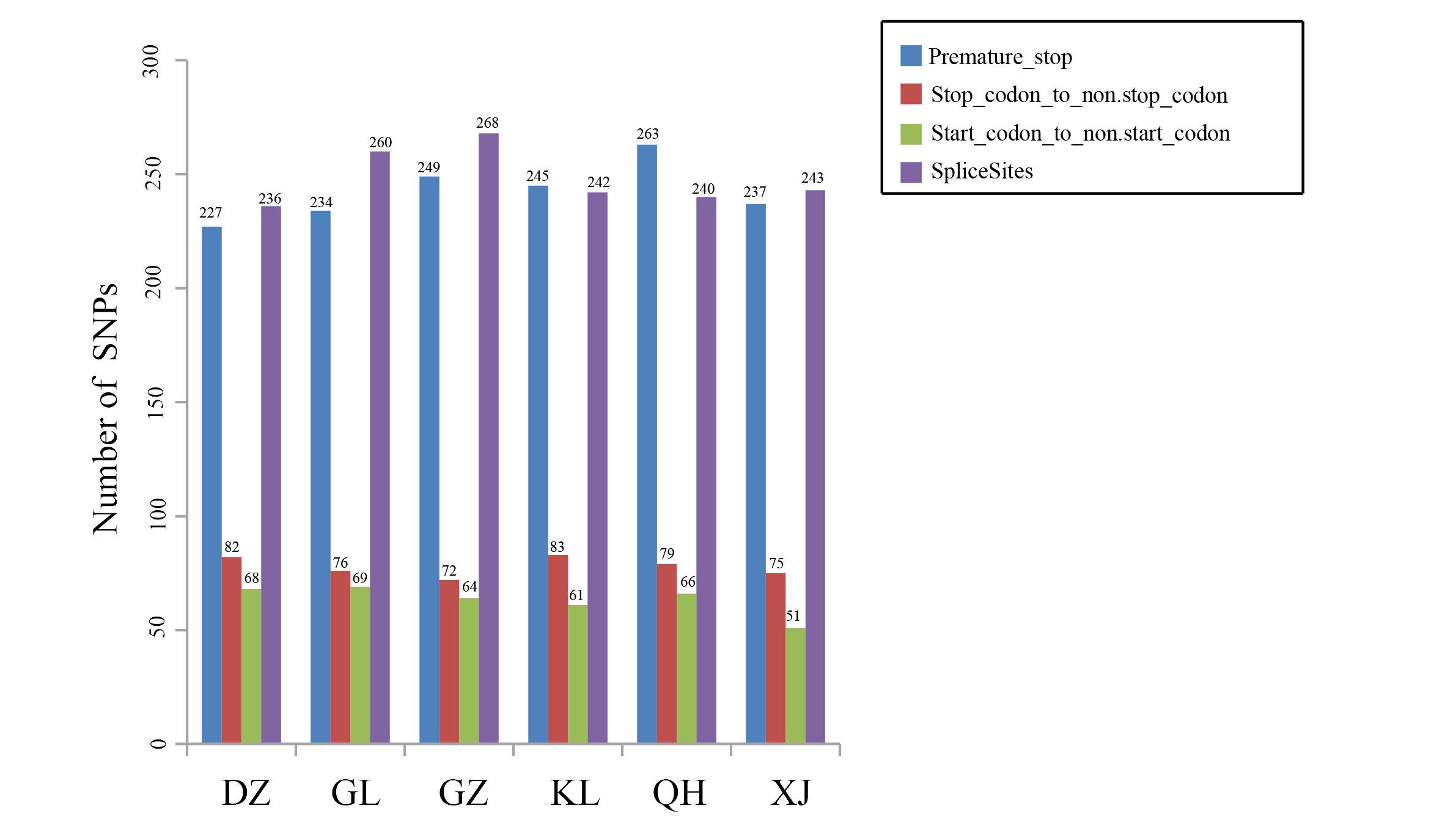

Supplement: Supplementary file 1 [file animals-10-01823-s001.zip › animals-929465-suppl/SupMaterial/S2.JPEG]
